# Application of through-the-scope twin clips for large defect closure after endoscopic submucosal tunnel dissection of a colonic laterally spreading tumor

**DOI:** 10.1055/a-2665-7420

**Published:** 2025-08-21

**Authors:** Wenqing Wu, Jianhuang Su, Jiayi Su, Chuntao Liu

**Affiliations:** 1 26455 Department of Gastroenterology, Beijing Friendship Hospital, Capital Medical University, State Key Laboratory for Digestive Health, National Clinical Research Center for Digestive Diseases, Beijing, China

Endoscopic submucosal dissection (ESD) is an effective endoscopic technique used to resect laterally spread tumors and early colorectal cancers. However, the large size of the lesion significantly increases the difficulty and risk of the procedure 1 . Previous studies have reported various endoscopic techniques to reduce resection difficulty in challenging cases, which include endoscopic submucosal tunnel dissection (ESTD), traction-assisted ESD, and other techniques. Additionally, the closure of large defects after ESD remains a technical challenge. This report presents a case involving the resection of a large laterally spreading tumor in the ascending colon via ESTD, followed by successful closure of the extensive defect using through-the-scope twin clips (TTS-TCs).

A 68-year-old woman presented with a laterally spreading tumor in the ascending colon. Preoperative contrast-enhanced abdominal computed tomography showed no enlarged lymph nodes. Colonoscopy revealed a large laterally spreading tumor in the ascending colon, which approximately involved three-fourths of the circumference ( Video 1 , Fig. 1 **a** ). According to a validated clinical score model 2 , it is “very difficult” with the score greater than 7 points. For difficult lesions, conventional ESD is time-consuming and has an increased risk of adverse events. Single-tunnel ESTD has been proven to be a time-saving procedure compared with conventional ESD. After submucosal injection of sodium hyaluronate solution, a positive lifting sign was observed ( Video 1 , Fig. 1 **b** ). First, the oral side of the lesion was cut transversely. Subsequently, a submucosal tunnel was created from the anal side to the oral side. After completion of the tunnel, the lateral mucosa was incised until complete removal of the tumor was achieved ( Video 1 , Fig. 1 **c** ).

**Fig. 1:**
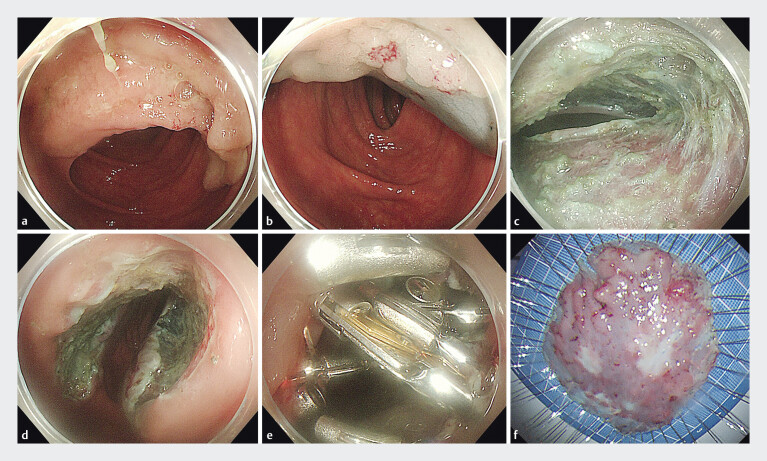
Procedure steps for application of through-the-scope twin clips for large defect closure after endoscopic submucosal tunnel dissection (ESTD) of a colonic laterally spreading tumor (LST). **a** An LST was located in the ascending colon. **b** Injecting fluid into the submucosa. **c** The ESTD defect located in the submucosal layer. **d** The defect extending to 3/4 of the colonic circumference. **e** Closure of the defect using through-the-scope twin clip and traditional clips. **f** The LST specimen.

The closure using TTS-TCs of a large ascending colon defect.Video 1

The lesion was successfully resected, with a mucosal defect size of approximately 5.9 cm × 6.4 cm, extending to 3/4 of the colonic circumference ( Video 1 , Fig. 1 **d** ). ESGE recommends prophylactic closure of resection defects ≥20 mm in size in the right colon, when closure is feasible 3 . Endoscopic defect closure techniques have been developed to reduce the incidence of postoperative adverse events. Through-the-scope clips (TTSCs) are used as a first-line strategy for closure of defects of no more than 2 cm. OTSCs may achieve adequate closure of larger defects of no more than 3 cm and full-thickness defects less than 2 cm in size. Endoscopic purse-string suture may be a preferred closure technique for larger defects, as the device is not limited by defect size; however, its use in the right colon may be limited 4 . Despite that, for lesions involving >3/4 of the luminal circumference, endoscopic purse-string suture carries significant risks of postoperative stenosis and is therefore not recommended.

TTS-TC is a promising tool for large-size mucosal defects closure under flexible endoscopy 5 . Therefore, we decided to use TTS-TCs to close the large defect. First, we clamped the oral side of the defect with one arm of the TTS-TC, then pulled the clip to the anal side, clamped the edge of the defect with the other arm, and deployed the TTS-TC. Considering the large size, the defect was closed along its longitudinal axis to achieve axial shortening, avoiding luminal stenosis. Two TTS-TCs were used to narrow the post-ESTD defect, which facilitated complete defect closure using conventional TTSCs ( Video 1 , Fig. 1 **e** ).

The patient was kept fasting for 72 hours. A prophylactic antibiotic was used. The patient was discharged uneventfully on day 8. TTS-TC is a precise, efficient, and safe device for endoscopic closure of large colonic defects.

Endoscopy_UCTN_Code_TTT_1AQ_2AK

## Notes

Endoscopy E-Videos https://eref.thieme.de/e-videos
*E-Videos* is an open access online section of the journal *Endoscopy* , reporting on interesting cases and new techniques in gastroenterological endoscopy. All papers include a high-quality video and are published with a Creative Commons CC-BY license. Endoscopy E-Videos qualify for HINARI discounts and waivers and eligibility is automatically checked during the submission process. We grant 100% waivers to articles whose corresponding authors are based in Group A countries and 50% waivers to those who are based in Group B countries as classified by Research4Life (see: https://www.research4life.org/access/eligibility/ ). This section has its own submission website at https://mc.manuscriptcentral.com/e-videos .
